# An Image Information-Based Objective Assessment Method of Technical Manipulation Skills for Intravascular Interventions

**DOI:** 10.3390/s23084031

**Published:** 2023-04-16

**Authors:** Jin Guo, Maoxun Li, Yue Wang, Shuxiang Guo

**Affiliations:** 1 School of Life Science, Beijing Institute of Technology, Beijing 100081, China; 2 China Academy of Electronics and Information Technology, Beijing 100041, China

**Keywords:** objective technical skill assessment, intravascular interventions, force and image data, machine learning

## Abstract

The clinical success of vascular interventional surgery relies heavily on a surgeon’s catheter/guidewire manipulation skills and strategies. An objective and accurate assessment method plays a critical role in evaluating the surgeon’s technical manipulation skill level. Most of the existing evaluation methods incorporate the use of information technology to find more objective assessment models based on various metrics. However, in these models, sensors are often attached to the surgeon’s hands or to interventional devices for data collection, which constrains the surgeon’s operational movements or exerts an influence on the motion trajectory of interventional devices. In this paper, an image information-based assessment method is proposed for the evaluation of the surgeon’s manipulation skills without the requirement of attaching sensors to the surgeon or catheters/guidewires. Surgeons are allowed to use their natural bedside manipulation skills during the data collection process. Their manipulation features during different catheterization tasks are derived from the motion analysis of the catheter/guidewire in video sequences. Notably, data relating to the number of speed peaks, slope variations, and the number of collisions are included in the assessment. Furthermore, the contact forces, resulting from interactions between the catheter/guidewire and the vascular model, are sensed by a 6-DoF F/T sensor. A support vector machine (SVM) classification framework is developed to discriminate the surgeon’s catheterization skill levels. The experimental results demonstrate that the proposed SVM-based assessment method can obtain an accuracy of 97.02% to distinguish between the expert and novice manipulations, which is higher than that of other existing research achievements. The proposed method has great potential to facilitate skill assessment and training of novice surgeons in vascular interventional surgery.

## 1. Introduction

Minimally invasive intravascular interventions have been widely adopted to diagnose and treat a variety of vascular diseases, such as stenosis, aneurysms, atherosclerosis, and thrombosis [1,2,3]. During intravascular interventional procedures, intravascular surgical tools (e.g., catheters, guidewires, stents, et al.) are percutaneously inserted in the vasculature and navigated to the desired treatment site within the cardiovascular system by two-degree-of-freedom (DoF) manipulations including pull/push in the axial direction and clockwise/counterclockwise twist in the radial direction. Compared with open surgery techniques, intravascular interventions have been associated with less blood loss, shorter recovery time, lower risk of infection, and less postoperative pain [4,5,6,7].

However, though intravascular interventions have brought many benefits and are being adopted worldwide, they are still associated with several challenges wherein steep learning curves for training surgeons in catheterization skills are of great concern [8,9,10,11]. Intravascular interventions are complex procedures that require surgeons to advance flexible and long catheters/guidewires at the proximal end within the fragile and complicated vasculature system with the lack of three-dimensional (3D) vascular anatomy visual feedback [12,13]. One of the main risks during intravascular interventions is frequent unintentional contact between catheters/guidewires and vascular walls, especially with diseased and weaken vessel walls, which has the potential for thrombosis, dissection, embolization, and perforation [14]. More serious is the risk of catastrophic consequences, including subsequent organ failure, fatal hemorrhage, and stroke [15]. The clinical success of intravascular interventions is highly dependent on the surgeon’s catheter/guidewire manipulation skills and strategies. The surgeon must possess a high level of catheterization skills before performing intravascular interventions. Therefore, it is essential to explore evaluation techniques that can accurately and objectively analyze catheter/guidewire manipulations performed by surgeons and assess their technical skills. 

Traditionally, written/oral examinations and case logs are employed to assess trainees’ surgical judgment and cognitive knowledge [16]. Additionally, trainees are asked to perform a set of predefined procedures on animals, cadavers, virtual simulators, or phantom models [17,18,19,20], and the assessments of their vascular interventional skills are carried out by supervising experts [21]. More standard and objective approaches are the application of endovascular global rating scales (GRS) and structured checklists [22,23], in which the predefined catheterization tasks are divided into a list of sequential subtasks. Supervising experts observe and manually score each subtask by examining trainees’ real-time manipulations or reviewing video recordings based on a list of technical criteria [24,25]. These approaches are the current gold standard for evaluating surgical competence, yet they have been associated with subjectivity and time consumption. The evaluation scores are assigned based on expert observation, which is inherently subjective to some extent and introduces bias. The GRSs generally contain a large number of criteria that are required to be graded manually by experts one at a time, making the scoring process quite burdensome and laborious.

In recent years, research efforts have been conducted to explore more objective and accurate approaches to evaluating vascular interventional skills with the aid of information technology. Some studies have explored objective evaluation techniques by analyzing the motions of catheters/guidewires during vascular interventional procedures. Rolls et al. [26] developed catheter-tracking software to conduct frame-by-frame motion analysis for fluoroscopic videos based on the vascular intervention surgical trainer (VIST) simulator. The two-dimensional catheter tip path lengths during interventional procedures performed by surgeons with varying experience were calculated and recorded as the performance metrics. The results indicated that the total path length correlated well with the surgeons’ skill levels and appeared shorter for highly experienced surgeons during identical carotid artery stenting procedures. It has been demonstrated that motion analysis of intravascular instruments derived from post hoc videos may be a valuable approach for the objective assessment of vascular interventional skills. Additional information derived from tracking data of catheters/guidewire, including average sub-movement duration, number of sub-movements, nondimensional jerks, average speed, the median value of procedure time, and movement smoothness, have been identified as potential performance metrics for evaluating catheterization skills [27,28,29,30]. An accuracy of 91% can be achieved to distinguish the attempts from expert and novice surgeons. The application of motion sensors attached to the tip of catheters has been another attractive approach to collecting data for distinguishing surgical skill levels during intravascular interventions. Schwein et al. [31,32] proposed a method to track the motion of catheters/guidewires by embedding electromagnetic (EM) sensors at the tip of both the leader and sheath. Tercero et al. [33,34] measured the surgeon’s hand and wrist motions in intravascular surgery simulation with three EM sensors, which were attached to the thumb and index fingers. The acquired data, combined with the motion data derived from an optical encoder, were investigated for technical skill evaluation. Srimathveeravalli et al. [35] also employed EM sensors to collect hand motion data for determining catheter/guidewire velocities and motion patterns of surgeons during interventional procedures. Zhou et al. [36] proposed an assessment framework for qualitative and quantitative evaluation of technical skills in percutaneous coronary intervention (PCI) based on natural behaviors including proximal force, hand motion, finger motion, and muscle activity. Three EM sensors attached to the surgeon’s fingertips of thumb and forefingers were used to collect hand motion data. Surface electrodes of electromyography (EMG) sensors were attached to the muscles, including biceps brachii, triceps brachii, abductor pollicis brevis, and dorsal interossei for recording muscle activities. Three-dimensional accelerometers were integrated with the above-mentioned EMG sensors and employed to determine the proximal force exerted on catheters/guidewires. Two fiber-optic bend sensors were introduced to acquire the bend curvatures of fingers. Finger motion was then derived from the obtained bend curvatures. Moreover, an additional EM sensor was connected to the guidewire tail by a piece of plastic tube to acquire guidewire movements. The obtained natural behaviors and motions were analyzed to classify the manipulations performed by novice and expert surgeons using the Gaussian mixture model and Mahalanobis distance. An accuracy of 92% can be achieved to distinguish between novice and expert attempts. In addition, a multilayer and multimodal-fusion architecture was developed to discriminate the manipulations made by surgeons with varying degrees of experience based on the aforementioned natural behaviors. An accuracy of 95% can be obtained to cluster the attempts performed by different skill-level groups [37]. Du et al. [38,39,40] proposed a random forest classification framework to properly identify the surgeon’s technical manipulation skills during PCI catheterization based on muscular activities and related hand motion, which were derived from physiological data detected by EM, sEMG, and flexible pressure sensors. The classification accuracy of 94.11% based on nineteen features from hand motion and muscle activities can be achieved. King et al. [41] developed a wireless sensor glove to overcome object occlusion and interference with hand motion by electrical wires. Eight sensors, including five 3-axis accelerometers, two 2-axis accelerometers, and a fiber-optic bend sensor, were used to acquire detailed gesture data for hand motion analysis. Another type of data that has been studied for technical skill assessment was the force and torque applied to catheters/guidewires during intravascular interventions. Rafii-Tari et al. [42,43,44] proposed a framework for objective technical skill assessment based on proximal force and torque data exerted on catheters/guidewires, contact force resulting from the tool–tissue interactions, and catheter tip motion. Force and torque data were measured by a custom-designed device, and the position information was acquired by an EM sensor integrated with the catheter tip. An accuracy of 90% can be obtained between the two skill levels. 

However, most of the existing objective assessment techniques are highly dependent on EM sensors, accelerometers, and EMG sensors. These sensors are required to be fixed to intravascular instruments (e.g., catheters, guidewires, et al.) and the surgeon’s upper limbs (e.g., hands, wrists, upper arms, et al.), which may influence the motion trajectory of intravascular tools and constrain the surgeon’s manipulations. In addition, incorrectly or inappropriately positioned sensors may affect the accuracy of data collection for skill evaluation.

Motivated by the above-mentioned drawbacks, we have developed a novel assessment framework to evaluate the surgeon’s manipulation skills. Nine objective performance metrics, including procedure time (PT), number of collisions between intravascular tools and vasculature (CN), path length (PL), number of speed peaks (SPN), slope variations of tool tip displacement (SV), vascular difficulty (VD), maximum force (MF), and mean force values (MFV), are proposed to assess the surgeon’s intravascular interventional skills, taking into consideration the traditional expert observation–based evaluation criterion. PT, CN, PL, SPN, SV, and VD are extracted and calculated from video sequences. MF and MFV are analyzed based on a 6-Dof force/torque sensor mounted under the vascular model. An SVM-based classification model is trained and used to discriminate the intravascular interventional skill levels of surgeons based on the above-mentioned performance metrics. The main contributions of this paper are as follows: (1) The proposed assessment framework disposes of the need to attach any sensors to the surgeon’s upper limbs and intravascular instruments. (2) During the data collection process, surgeons are allowed to drive the catheters/guidewires within the vascular model with the same manipulation skills that they utilize in real intravascular interventions. (3) Nine objective performance metrics are investigated for technical skill evaluation, in which the number of collisions, slope variations, and the number of speed peaks, to the best of our knowledge, have rarely been exploited.

## 2. Materials and Methods

### 2.1. The Proposed Framework for Catheterization Skills Assessment

**Figure 1:**
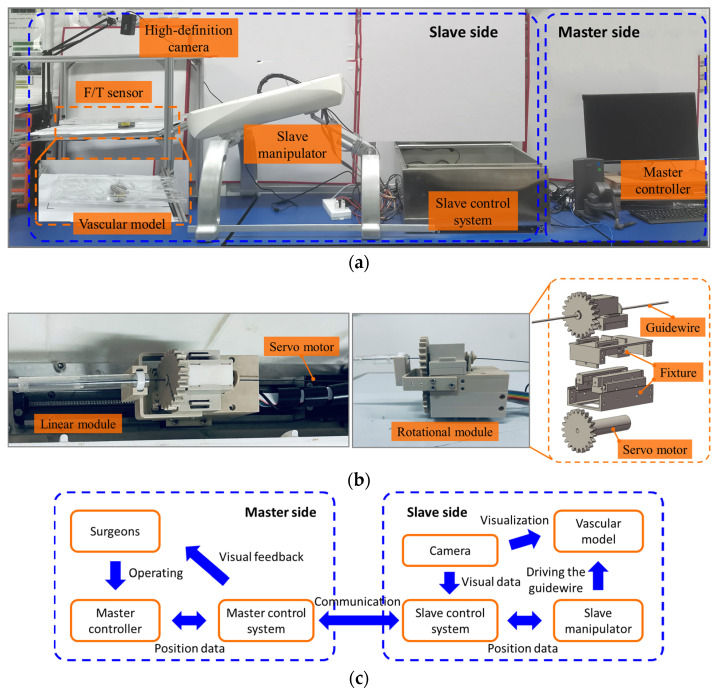
The framework for catheterization skill assessment: (**a**) the experimental setup for data collection; (**b**) the rotational and linear modules of the slave manipulator; (**c**) a schematic diagram of the master-slave vascular interventional surgical robot.

The assessment framework is designed to accurately acquire the motion data of intravascular instruments from the vascular model, contact force data between instruments and the vascular model, and the manipulation data from the surgeon’s performance, as illustrated in Figure 1a.

A remote-controlled vascular interventional surgical (VIS) robot [45,46] is used to drive catheters/guidewires within the vascular model. The VIS robot consists of the master side and the slave side. On the master side, a 6-DoF, commercially available device, (Geomagic Touch, 3D Systems Corp, Rock Hill, SC, USA) is employed as the master controller. The handle of the master controller is extremely light and flexible, without placing any additional strain on the operator’s hands. Intravascular interventional manipulation by surgeons generally involves three patterns: push and pull in axial manipulation for insertion and withdrawal of the intravascular tool, clockwise and counterclockwise twist in circumferential manipulation to control the orientation of the tool tip when encountering vascular bifurcations, and combined manipulation to simultaneously adjust the position and orientation of the tool. Surgeons are allowed to apply the same manipulations on the handle of the master controller in a comfortable state, for the purpose of providing a more immersive feel during data collection. The master control system is used to record the surgeon’s manipulation data and transmit these data to the slave side. On the slave side, an anthropomorphic, transparent vascular model of the aortic arch with left subclavian artery, left common carotid artery, brachiocephalic artery, and ascending and descending aorta based on real human data is employed to simulate the vasculature of patients. A high-definition camera, positioned on top of the vascular model, is used to provide visualization of the vascular model at a rate of 20 frames per second. It is employed to simulate X-ray fluoroscopy and provide two-dimensional (2D) visual feedback for the surgeon. Additionally, the camera is responsible for recording videos of catheter/guidewire motion during catheterization tasks. A 6-DoF force/torque sensor (ATI Gamma SI-65-5, ATI Industrial Automation, Inc., Apex, NC, USA) is rigidly mounted under the vascular model, which can provide force and torque data in all three directions (X, Y, and Z). The slave manipulator is capable of clamping and manipulating the catheter or guidewire by replicating the same manipulations from the master side. As illustrated in Figure 1b, a motorized linear module was employed to insert or withdraw the catheter/guidewire within the vascular model, and a motorized rotational module was used to twist the catheter/guidewire. The controller area network (CAN) bus was adopted for communication between the master side and the slave side. The schematic diagram of the master-slave vascular interventional surgical robot is illustrated in Figure 1c.

### 2.2. Participants and Catheterization Tasks

Six intravascular surgeons were recruited during data collection. Participants were divided into two groups, the expert group and the novice group, based on their experience in vascular interventional surgery. The expert group involved three expert surgeons who have performed more than 200 intravascular interventional procedures and the novice group included three novice surgeons who have performed fewer than 20 interventions. All the surgeons who participated in this study had the right dominant hand. Prior to data collection, surgeons were trained to familiarize themselves with the operation of the master controller and the catheterization tasks in advance. After the data collection started, the surgeon’s manipulations were allowed to proceed without intervention.

**Figure 2:**
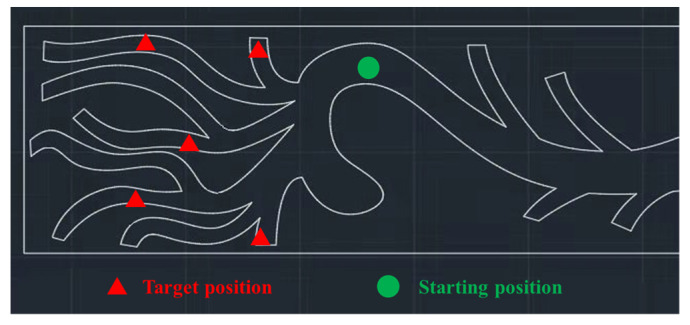
The five vascular interventional tasks for assessing surgeons’ guidewire manipulation skills.

The focus of this study is the interventional procedure of delivering the medical guidewire from the descending aorta, passing through the aorta arch to the brachiocephalic artery, left common carotid artery, and left subclavian artery, respectively. This procedure is a highly demanding test of the surgeon’s familiarity with the anatomical structures of the vessels and the surgeon’s skills in guidewire manipulation. Varying levels of intravascular interventional skills often result in varying surgical outcomes. Thus, five tasks were designed to evaluate the surgeon’s guidewire manipulation skills. As illustrated in Figure 2, surgeons were asked to drive the guidewire from the descending aorta (at the position marked by the green triangle) to the brachiocephalic artery, left common carotid artery, and left subclavian artery (at the position marker by the green rectangles). The procedure of delivering the guidewire to each target position was repeated five times, giving a total of one hundred and fifty trials. To guarantee the consistency of manipulation, all trials in these five catheterization tasks were implemented at the same initial position and orientation of the guidewire. To avoid physical fatigue, surgeons were allowed to rest for 3–4 min between different trials. 

### 2.3. Image Processing Algorithm

**Figure 3:**
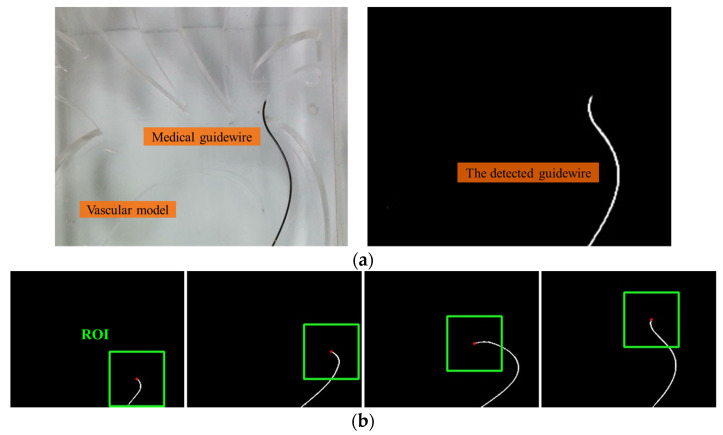
The image processing algorithm for the guidewire detection: (**a**) the captured guidewire within the vascular model based on the image processing algorithm; (**b**) the detected guidewire tip.

To evaluate the trajectory of the input guidewire in a quantitative way, an image processing algorithm based on the OpenCV library in Python is proposed to automatically detect and track the guidewire tip frame by frame within the vascular model. This algorithm provides the 2D position, in pixel coordinates, of the catheter tip within the recorded videos. Each frame of the video is converted into a binary image using a cut-off threshold to extract the shape of the guidewire. Pixels with a greyscale value below the threshold are assigned the value ‘0′, while those above are assigned the value ‘1’. The shape of the guidewire is delineated by the white pixels in the resulting binary image (as shown in Figure 3). The position of the guidewire tip is determined by the Shi-Tomasi corner detection algorithm. The algorithm is extremely efficient in locating the position of the guidewire tip in the image coordinate system. To reduce processing time and improve tracking reliability, after the initial tip position is detected in the first frame, a region of interest (ROI) is automatically generated around the guidewire tip for subsequent frames. In our experiments, the accuracy of the proposed image processing method can exceed 99.7%. The failed detection of the guidewire tip in the first frames has not occurred. This is because the guidewire is stationary in the first frames of the video recordings and the guidewire can be easily separated from the rest of the scene. The image processing method failed to detect the wire tip only in a few cases while the guidewire was being manipulated. In this situation, the sequence number of the frame can be recorded and the position of the wire tip in these frames can be manually located.

### 2.4. Objective Performance Metrics

Taking full account of the scoring criteria in the traditional gold standard for assessing surgical competence, three new performance metrics are proposed, including the number of speed peaks, number of collisions, and slope variations, to quantify the experience of supervising experts. In addition, two performance metrics related to force data during intravascular interventions, including maximum force and mean force values, and three typical metrics, including path length, procedure time, and vascular difficulty, are integrated to form the overall assessment system.

#### 2.4.1. Path Length

**Figure 4:**
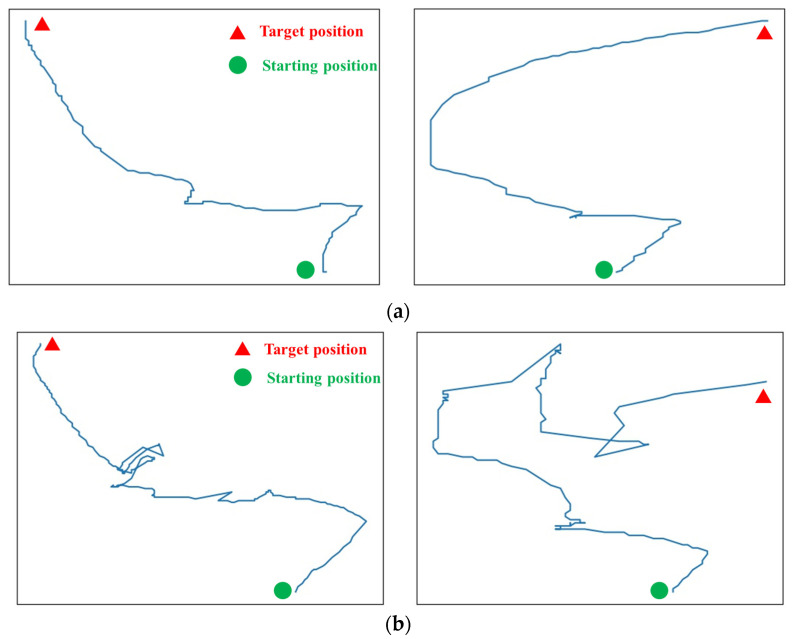
The motion trajectory of the guidewire tip during two intravascular tasks performed by (**a**) expert surgeons and (**b**) novice surgeons, respectively.

It has been demonstrated that the total path length of the tip of the catheter correlated with the manually scored GRS. For highly experienced surgeons, the path length of the catheter’s tip appeared shorter during intravascular interventions. This is because experienced surgeons are more likely to succeed with fewer attempts to deliver catheters/guidewires to the target vessel. Novice surgeons, due to their unfamiliarity with vascular anatomy or poor intravascular manipulation skills, are more likely to attempt catheter/guidewire delivery more times during interventional procedures, resulting in longer trajectories (as illustrated in Figure 4). Therefore, in this study, the path length for the interventional task is introduced as the performance metric. The total path length of the tip of the guidewire for each interventional task was calculated by (1).

(1) $$the\;L=\overset{n-1}{\underset{i=1}{\sum }}\sqrt{{\left(x_{i+1}-x_{i}\right)}^{2}+{\left(y_{i+1}-y_{i}\right)}^{2}}$$

where *L* indicates the total path length, and $x_{i}$ and $y_{i}$ represent the horizontal and vertical coordinates of the position of the guidewire tip in the *i*-th frame of the recorded video, respectively. 

#### 2.4.2. Number of Collisions between Catheters/Guidewires and the Vascular Model

In order to reach the target treatment site of the vasculature, intravascular interventions require complex maneuvering of catheters/guidewires. The inadvertently frequent contact between catheters/guidewires and vessel walls is one of the major risks of damaging the patient’s blood vessels. In this study, the number of collisions between the input guidewire and the vascular model was innovatively added as one of the performance metrics to evaluate intravascular skills. We defined the guidewire-vessel collision in the recorded videos as the situation where the tip of the guidewire touched the vessel wall and the rest of the guidewire was not in contact with the vessel wall in the current frame, and in the next frame, the catheter tip remained in contact and stationary with respect to the vessel wall while the rest of the guidewire changed position or morphology. In this situation, the guidewire may be accumulating strength, which leads to an immediate change in position within the vasculature and potential damage to the vessel wall. Apart from a few exceptional cases, experienced surgeons tend to avoid collisions during intravascular interventions through their technical skills.

#### 2.4.3. Number of Speed Peaks

**Figure 5:**
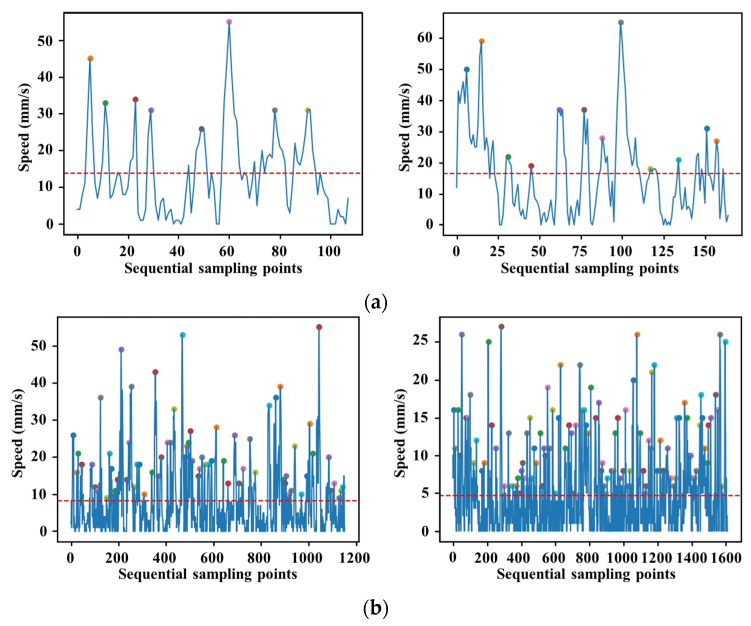
The detected speed peaks during two intravascular tasks performed by (**a**) an expert surgeon and (**b**) a novice surgeon, respectively.

Performance metrics related to smooth catheter navigation without unnecessary movements are included in almost all established intravascular GRSs and checklists. It was demonstrated that movement-based evaluation criterion quantifying smoothness was demonstrated to have a stronger correlation with the structured grading assessment than metrics that simply quantify the kinematics of the catheter tip [28]. In contrast to existing smoothness evaluation methods, the number of speed peaks and slope variations in the motion trajectory of the guidewire tip were employed as the performance metrics to describe the smoothness of guidewire navigation in this study. The number of speed peaks represents the number of accelerating and decelerating manipulations. Fewer peaks are associated with smoother movements. In our case, data on the speed profile of the guidewire movement were extracted from the master controller. The current speed value can be identified as a candidate speed peak if it is greater than all five speed values before and after it. To eliminate small speed fluctuations, a candidate speed peak was finally identified as the speed peak if it was greater than the average speed value of the current intravascular task. The speed peak can be computed by (2).

(2) $$v_{peak}=\underset{\left(i-5,i+5\right)}{\mathrm{Max}}\left(v_{i}\right)\cap \left(v_{i}>v_{mean}\right),\;i\in \left(6,\;n-5\right)$$

where $v_{mean}$ represents the average speed of the guidewire motion in the current intravascular task. Figure 4 illustrates the motion trajectory of the guidewire tip during two intravascular tasks performed by expert surgeons and novice surgeons. There is a significant difference in the smoothness of the guidewire tip trajectory within the vascular model between expert and novice surgeons. The trajectory, generated from the expert surgeon’s manipulation data, is significantly smoother and corresponds to a smoother manipulation, whereas the trajectory from the novice surgeon is more chaotic. The novice surgeon tends to have a higher failure rate when delivering the guidewire through the vessel bifurcation. The novice surgeon often re-delivers the guidewire after withdrawal and twisting manipulations, resulting in a messy trajectory. Accordingly, the number of speed peaks detected during the intravascular tasks performed by the expert surgeon is significantly lower than that of the novice surgeon, as illustrated in Figure 5.

#### 2.4.4. Slope Variations of Tool Tip Displacement

**Figure 6:**
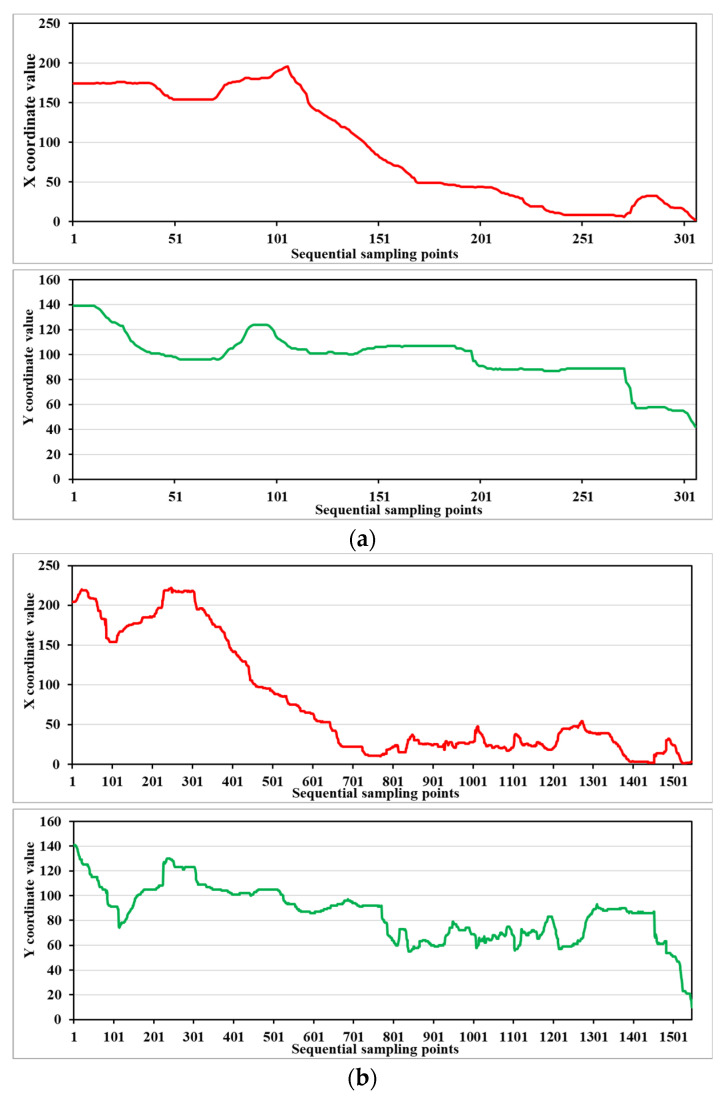
The slope variations of the guidewire tip in X and Y directions during one intravascular task performed by (**a**) an expert surgeon and (**b**) a novice surgeon, respectively.

In addition to the speed peaks, variations in the slope of the trajectory of the guidewire tip were innovatively used as another performance metric to describe the smoothness. The position of the guidewire tip was detected frame by frame from the recorded videos. The horizontal and vertical coordinates of the guidewire tip in the image coordinate system were recorded. Figure 6 illustrates the variations in the horizontal and vertical coordinates of the guidewire tip trajectory during an intravascular task performed by expert and novice surgeons, respectively. It can be seen that the variation trends of the curves in the horizontal and vertical coordinates are approximately the same between the skill groups, indicating that the path of the guidewire is similar for the same target position. However, there is a clear difference in the smoothness of the curves between the two skill groups. The curves generated from the guidewire tip trajectory performed by experienced surgeons are significantly smoother. The accumulated variations in the slope of the guidewire tip trajectory can be calculated using the following equation.

(3) $$\{\begin{array}{c} kx_{m}=\left|x_{m+1}-x_{m}\right|\\ ky_{m}=\left|y_{m+1}-y_{m}\right| \end{array}\;,\;\;\;\;\;m\in \left(2,\;n-1\right)$$

(4) $$\{\begin{array}{c} \Delta kx=\sum_{i=1}^{n-1}kx_{i}\\ \Delta ky=\sum_{i=1}^{n-1}ky_{i} \end{array}$$

where $x_{m}$ and $y_{m}$ represent the horizontal and vertical coordinate values in the image coordinate system, $kx_{m}$ and $ky_{m}$ indicate the variations between two neighboring positions in X and Y directions, and Δkx and Δky represent the accumulated variations in the slope.

#### 2.4.5. Procedure Time

The duration of intravascular interventional surgery determines the time of X-ray exposure for both the surgeon and patient, which is harmful to their physical health. The longer the vascular interventional procedure takes, the more fatigued the surgeon becomes, which often reduces the safety of the procedure. Experience surgeons show consistently lower values of procedure time than novices because they tend to be more familiar with the anatomy of the human vasculature and are better equipped with the technical skills to manipulate intravascular interventional instruments. Therefore, the procedure time for each task can be used as the performance metric to evaluate the surgeon’s intravascular skills. The procedure time for each task was extracted from the recorded videos. 

#### 2.4.6. Maximum Force and Mean Force Values

**Figure 7:**
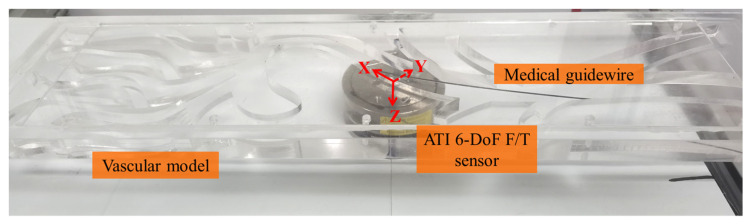
The configuration of the 6-DoF F/T sensor to acquire the contact forces generated from the interaction between intravascular tools and the vasculature.

In practice, surgeons also rely on tactile cues, in addition to visual cues, to guide catheters/guidewires to the desired treatment site within the patient’s vasculature. Excessive force on the blood vessel walls applied by the surgeon may injure and even rupture the vessel, causing irreparable damage to the patient. The reduction in contact forces has the potential to lower intraprocedural risks for diseased vessel walls. Experienced surgeons often pay extra attention to this issue during interventional procedures. The maximum and mean forces obtained from the intravascular interventional tasks performed by experienced surgeons tend to be lower than the forces obtained from the procedures performed by novices. The contact forces generated from the interaction between intravascular tools and the vasculature were measured using the ATI 6-DoF F/T sensor, as illustrated in Figure 7. 

The mean force value and maximum force during the intravascular interventional tasks were employed as the performance metrics to differentiate expert and novice performance, which can be calculated by (5) and (6):

(5) $$F_{mean}=\frac{\sum_{i=1}^{n}\sqrt{F_{x}^{2}+F_{y}^{2}+F_{z}^{2}}}{n}$$

(6) $$F_{max}=\underset{i\in \left(1,n\right)}{\mathrm{Max}}\left(\sqrt{F_{x}^{2}+F_{y}^{2}+F_{z}^{2}}\right)$$

where $F_{x}$, $F_{y}$ and $F_{z}$ are the component forces in the X, Y and Z directions, respectively.

#### 2.4.7. Vascular Difficulty

Vascular difficulty is the degree of surgical difficulty in terms of the impact of the vascular structure on the surgeon’s operation. It depends on the complexity of the physiological structure of the blood vessels. A higher degree of vascular difficulty indicates a more complex vascular structure that is difficult to treat during vascular interventional surgery. In the GRS for intravascular interventional surgery, the degree of difficulty was considered to be a measurable scale [28]. Vascular difficulty at the aortic arch can be objectively determined by the diameter of the vessel branches, the distance between the target vessel branch and the descending aorta, and the angle of inclination between the target vessel branch and the cut surface of the aortic arch [11]. The vascular difficulty is an important objective measure in assessing the surgeon’s technical skills. For example, the difference in manipulation data between expert and novice surgeons is not significant when delivering the guidewire within the vascular branch with low vascular difficulty, because expert surgeons do not have to use their dexterity skills. Therefore, vascular difficulty is combined as a performance metric in this study.

## 3. Results

### 3.1. Statistical Analysis

Statistical analysis was performed to analyze the contribution of the proposed performance measures to the intravascular skill assessment model. The t test, an inferential statistic used to determine whether there is a significant difference, was used to test the significance of the differences between the manipulation data for each indicator obtained from the same skill group at different vascular difficulty levels. A *p*-value less than 0.05 was considered statistically significant. The results of the significant difference analysis are shown in Table 1. 

For experienced surgeons, there were significant differences in the number of collisions between the guidewire and the vascular model and the variation of the slope in the vertical direction when performing intravascular interventions within vessel branches with different vascular difficulties. On the other hand, the variability was not significant for the metrics of the procedure time, the maximum and mean force applied to the vascular model, and the number of speed peaks. The variability of the manipulation data for these metrics is not significant because experienced surgeons are able to control the contact force between the guidewire and the vascular model well and are more familiar with the anatomical structure of the vasculature, even when performing on different vessels with different vascular difficulties. For novice surgeons, there were significant differences in the slope variations in both the horizontal and vertical directions, which were used as two performance metrics of trajectory smoothness when performing on different vessels with different vascular difficulties.

Similarly, we also verified the variability between the manipulation data of different skill groups for the same vascular difficulty, and the results are shown in Table 2. It can be seen that a significant difference exists in the performance metrics of the number of collisions and the path length when performing on the vessels with the lower vascular difficulty level between the different skill groups, and there is a significant difference in the metrics of the number of speed peaks, procedure time, and mean force when operating on the vessels with higher vascular difficulty. The performance metrics used in this study have varying degrees of contribution to the evaluation model. It is worth noting that the proposed performance metrics, including the number of collisions, the number of speed peaks, and the slope variations in X and Y directions, showed a significant difference in the manipulation data performed by different skill groups or on the vessels with different vascular difficulties.

### 3.2. SVM-Based Assessment Model

The binary SVM classifier with radial basis function (RBF) kernel was used to classify the manipulations performed by novice and expert surgeons. The nine features based on the performance metrics during the intravascular interventional tasks were extracted to construct the feature space for the binary SVM classification. The feature vectors, combined with the corresponding class labels, were used to train the SVM classifier, as shown in (7):

(7) Input=[I, class labels]

where *I* represent the vectors including nine feature values, and *class labels* indicate whether the surgeons are from the expert group or the novice group. 

Proper training is a critical step in supervised machine-learning modeling. The SVM classification maps the input feature vectors into a higher dimensional space in a nonlinear way and determines a separating hyperplane with the maximum margin between the two classes using an optimization step. The performance of the SVM-based assessment model was evaluated using the k-fold cross-validation method. The data set was divided into a training set containing 65% of the total number of data samples, while the remaining 35% was used to test the model. After dividing the data into the training set and the verification set, the SVM model was trained and the classification accuracy was calculated. To obtain the optimal model, the parameters for the SVM model training should be adjusted in real time. Among these parameters, the kernel function and the regularization parameter have a significant impact on the model. The bigger the regularization parameter, the more penalty SVM gets when it makes a misclassification. Setting a small regularization parameter value can reduce penalties for misclassification and allows for fault tolerance owing to strong generalization capabilities. The complexity of the model would be altered by adjusting the regularization parameter. The radial basis function kernel, which is suitable for moderate sample sizes and small numbers of features, was used to map the sample space to infinite dimensional space after parameter adjustment. After several training sessions, the regularization parameter was adjusted to 0.8 and the gamma was adjusted to 20 to obtain higher accuracy. The SVM-based assessment model based on nine performance metrics can achieve an accuracy of 97.02% in discriminating expert and novice manipulations.

## 4. Conclusions

Assessment of surgical skills is an important part of the surgeon’s professional development. Moreover, in light of major surgical errors, the maintenance of surgical competence has been a matter of great public concern. However, there is currently no standardized way of assessing vascular interventional skills. Traditionally, the trainee to be assessed performs a series of predefined procedures while being supervised by experts in the field. The supervising experts subjectively assess performance on the basis of the trainee’s manipulation in accordance with the GRS and checklists. The need for reliable and objective techniques to assess the surgeon’s intravascular skills, especially during complex interventional procedures involved in vascular interventional surgery, has become one of the main priorities of the surgical community. Motion sensors attached to the tip of intravascular instruments or the surgeon’s upper limbs have been the most commonly used approach to collect data to objectively differentiate surgical skill levels during intravascular procedures. However, the added sensors may influence the physical properties of intravascular tools and constrain the surgeon’s manipulations. Additionally, incorrectly or inappropriately positioned sensors may influence the accuracy of data collection for skill evaluation. To overcome these drawbacks, we developed a novel assessment method to evaluate the surgeon’s manipulation skills based on nine objective performance metrics. During the data collection process, surgeons are allowed to manipulate the catheters/guidewires within the vascular model with the same manipulation skills that they use during real intravascular interventions, without the need to attach any sensors. An SVM-based assessment model is proposed to discriminate between expert and novice manipulation, with a classification accuracy of 97.02%, which is higher than other existing research results. In this study, we have not split the data by participant. In our future work, more surgeons will be recruited for data collection and some other algorithms (e.g., deep learning techniques) and multiple validation methods will be investigated.

## Figures and Tables

**Table 1:** The significance of the differences between the manipulation data for each indicator obtained from the same skill group at different vascular difficulty levels (*p*-value).

Performance Metrics	Expert Surgeons	Novice Surgeons
Path length	0.005	0.044
Number of collisions	0.021	0.070
Number of speed peaks	0.232	0.089
Slope variations in X direction	0.065	0.044
Slope variations in Y direction	0.023	0.002
Procedure time	0.425	0.055
Maximum force	0.305	0.154
Mean force	0.407	0.405

**Table 2:** The significance of the differences between the manipulation data of different skill groups for the same vascular difficulty (*p*-value).

Performance Metrics	Vessels with Lower VD	Vessels with High VD
Path length	0.046	0.080
Number of collisions	0.033	0.080
Number of speed peaks	0.095	0.030
Slope variations in X direction	0.161	0.303
Slope variations in Y direction	0.128	0.266
Procedure time	0.132	0.043
Maximum force	0.088	0.198
Mean force	0.222	0.049

## Data Availability

The data that support the findings of this study are available from the corresponding author upon reasonable request.
